# Hydrostatic Pressure Sensing with High Birefringence Photonic Crystal Fibers

**DOI:** 10.3390/s101109698

**Published:** 2010-11-01

**Authors:** Fernando C. Fávero, Sully M. M. Quintero, Cicero Martelli, Arthur M.B. Braga, Vinícius V. Silva, Isabel C. S. Carvalho, Roberth W. A. Llerena, Luiz C. G. Valente

**Affiliations:** 1 Pontifical Catholic University of Rio de Janeiro, Rua Marquês de São Vicente 225, 22453-900, Rio de Janeiro, RJ, Brazil; E-Mails: fc.favero@fis.puc-rio.br (F.C.F.); sully@aluno.puc-rio.br (S.M.M.Q); vinicius_v_silva@hotmail.com (V.V.S.); isabel.carvalho@puc-rio.br (I.C.S.C.); roberan@puc-rio.br (R.W.A.L); luizguedes@puc-rio.br (L.C.G.V.); 2 Department of Electronics, Federal University of Technology-Parana, Av Monteiro Lobato, s/n–km 04-Ponta Grossa, PR, 84016-210, Brazil; E-Mail: cmartelli@utfpr.edu.br

**Keywords:** photonic crystal fiber, high birefringence, hydrostatic pressure sensing, air-silica structured fiber, microstructured fiber

## Abstract

The effect of hydrostatic pressure on the waveguiding properties of high birefringence photonic crystal fibers (HiBi PCF) is evaluated both numerically and experimentally. A fiber design presenting form birefringence induced by two enlarged holes in the innermost ring defining the fiber core is investigated. Numerical results show that modal sensitivity to the applied pressure depends on the diameters of the holes, and can be tailored by independently varying the sizes of the large or small holes. Numerical and experimental results are compared showing excellent agreement. A hydrostatic pressure sensor is proposed and demonstrated using an in-fiber modal interferometer where the two orthogonally polarized modes of a HiBi PCF generate fringes over the optical spectrum of a broad band source. From the analysis of experimental results, it is concluded that, in principle, an operating limit of 92 MPa in pressure could be achieved with 0.0003% of full scale resolution.

## 1. Introduction

The sensitivity of high birefringence (HiBi) fibers to hydrostatic pressure has interested the scientific community as a feasible alternative for pressure sensing. The anisotropic nature of the core region, including stress distribution and geometry, makes HiBi fibers sensitive to axially symmetric transverse forces acting on the external surface of the fiber. HiBi photonic crystal fibers, where the degeneracy is lifted by a break in structural symmetry, present some important advantages over conventional stress induced HiBi optical fibers, and have recently been employed to demonstrate pressure sensors based on either intermodal interferometry [1–4] or Type II fiber Bragg gratings [5].

Conventional all-solid HiBi fibers present residual stresses that are largely dependent on material thermal relaxation, and consequently are highly sensitive to temperature variations. Temperature sensitivity in PCFs, on the other hand, is mostly associated to the fiber thermo-optic coefficient and can also be made negligible [6]. They can be specifically tailored to enhance their response to hydrostatic pressure while presenting negligible temperature dependence [7]. The internal microstructure of holey PCFs can be engineered to behave as an air-silica composite material, where size of holes and their distribution at the core and cladding regions of its cross section can increase or decrease response to strain, pressure, or temperature [8,9]. Another advantage concerning pure silica PCFs lies in their chemical resistance to hydrogen, which makes them particularly attractive for sensing applications in high temperature and hydrogen rich environments. The absence of defects induced by germanium or phosphorous doping in the glass matrix greatly enhances the fiber immunity to H_2_ diffusion and reaction, which are both responsible for decreasing the fiber’s transparency and mechanical strength [10].

The work presented here brings detailed information on the effect of hydrostatic pressure over the waveguiding properties of PCFs and also insight on ways of shaping the fiber sensitivity. Given the numerous design possibilities to achieve high birefringence with PCFs, a fiber design that is already available in the market was chosen as starting point and reference. Multiphysics finite element analysis is employed to numerically evaluate the coupled mechanical and optical response of HiBi PCFs to hydrostatic pressure. With the aid of a numerical model, we analyze in detail the changes in modal birefringence brought about by slight modifications in the design of the reference fiber. This same reference PCF is then employed to demonstrate a hydrostatic pressure sensor based on polarization mode interference within the fiber. Finally, numerical and experimental results are compared showing excellent agreement.

## 2. HiBi PCF Fiber Design and Numerical Modeling

**Figure 1.**
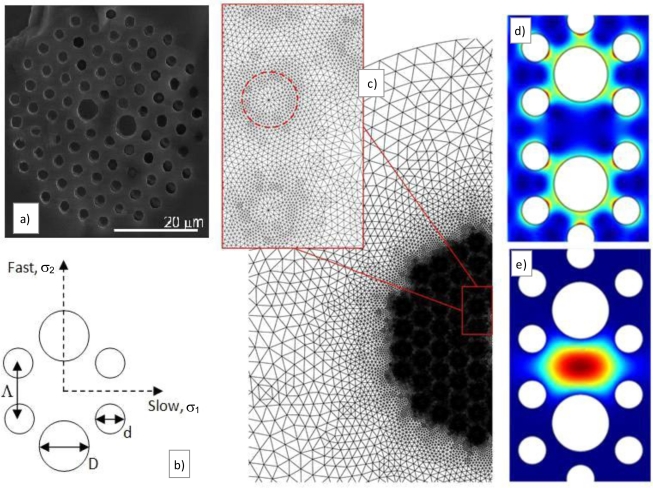
**(a)** Scanning electron microscopy picture of the pure silica HiBi PCF fiber used as reference for the numerical modeling and demonstration of a hydrostatic pressure sensor; **(b)** schematic representation of the fiber core showing the fast and slow axis as well as the stress components (*σ*_1_ and *σ*_2_); (**c**) mesh representing the fiber structure for the finite element analysis–inset: zoom in on the structured region defining the fiber core; **(d)** stress distribution (*σ*_2_ component) across the fiber core; and **(e)** numerically calculated electric field distribution for one polarization eigenstate mode.

There are distinct ways of tailoring PCFs to enhance their birefringence. This may be accomplished, for instance, by breaking the n-fold rotational symmetry of an otherwise n-fold symmetric holey microstructure [11]. In another approach, elliptical holes with equal or different sizes have been employed to define both the cladding and core regions of the PCF [12,13]. Birefringence as high as of the order of 10^−2^ can be achieved in this fashion. However, only a few of these fibers are commercially available at this time. Therefore, in this paper, we have resorted to a commercial HiBi PCF supplied by NKT Photonics. Their fiber model PM-1550-01 [14] was used here as a reference for numerical and experimental investigations. This same fiber has been employed elsewhere to demonstrate pressure [1–4], strain [15], torsion [16], and magnetic sensors [17]. The cross-section of the fiber PM-1550-01, depicted in Figure 1(a), contains a hexagonal lattice of small holes defining the cladding region. A missing hole in the middle gives rise to a solid core. Due to the high index contrast between core and cladding regions, the guidance mechanism is predominantly governed by total internal reflection. The distance between holes forming the periodic lattice is *Λ* = 4.4 μm. The diameter of the small air holes is *d* = 2.2 μm. Birefringence is generated by replacing two of the small holes in the innermost ring surrounding the core by larger holes of nominal diameter D = 4.5 μm. The solid portion of the fiber is made of pure silica (n_SiO2_ = 1.45). In the numerical study reported here, we have analyzed geometrical variations of this reference PCF where *d* and *D* were either enlarged or reduced.

The stress distribution within the fiber and the propagation constants of the two confined modes under the action of hydrostatic pressure are calculated using the finite element code COMSOL Multiphysics^®^ (version 3.5) [18]. As highlighted in Figure 1(b), the mesh density of the finite element model is higher near the structured region of the fiber cross-section. Satisfactory refinement was obtained after a convergence analysis lead to a mesh with 153,280 triangular elements. Figure 1(d,e) present examples of the stress and electric field distributions within the waveguide calculated using the finite element software.

The first step in order to assess the effect of hydrostatic pressure in the waveguiding properties of the PCF is a numerical evaluation of the stress distribution in the pressure loaded fiber. A state of plane-strain was assumed in the simulations, and the values employed for the silica glass’ Young modulus and Poisson ratio were 72.5 GPa and 0.17 respectively. After obtaining the stress field due to the applied pressure, the new refractive index distribution in the fiber cross-section is evaluated by using the stress-optical relation [19]:

(1) $$\begin{array}{c} n_{1}=n_{0}-C_{1}\sigma_{1}-C_{2}(\sigma_{2}+\sigma_{3})\\ n_{2}=n_{0}-C_{1}\sigma_{2}-C_{2}(\sigma_{1}+\sigma_{3})\\ n_{3}=n_{0}-C_{1}\sigma_{3}-C_{2}(\sigma_{1}+\sigma_{2}) \end{array}$$

where *σ*_1_, *σ*_2_, and *σ*_3_ are the principal stresses in the fiber, while *C*_1_ and *C*_2_ correspond to the stress-optical constants, which, for silica glass, are 0.69 × 10^−12^ and 4.2 × 10^−12^ Pa^−1^ in that order.

The new refractive index distribution, calculated for the pressure loaded fiber through Equation (1), is then used to numerically obtain the effective indices of the two orthogonally polarized fundamental modes (LP_01-slow_ and LP_01-fast_). The modal fields and effective indices are calculated using a full-vectorial model and the Maxwell’s differential equation is expressed in terms of transverse electric and magnetic fields. In the numerical model, instead of resorting to a perfecly matching layer boundary condition, we have assumed a perfect magnetic conductor condition along the fiber’s outermost boundary. This simpler condition could be employed here due to the fact that we are interested in simulating only the two fundamental LP_01_ modes, whose fields rapidly decay towards the computational boundary. Furthermore, these modes present negligible loss within the fiber lengths employed in the present investigation.

Phase and group modal birefringence, denoted as *B* and *G* respectively, are given by [6,7]:

(2) $$B=n_{\mathrm{LP}01}^{\mathrm{slow}}-n_{\mathrm{LP}01}^{\mathrm{fast}}$$

(3) $$G=B-\lambda \frac{\mathrm{dB}}{d\lambda }$$

where $n_{\mathrm{LP}01}^{\mathrm{slow}}$ and $n_{\mathrm{LP}01}^{\mathrm{fast}}$ are the effective refractive indices of the two polarization modes and *λ* the wavelength.

### 2.1. Results and Discussion

The coupled elasto-optic response in the presence of hydrostatic pressure was numerically investigated for two groups of fibers. In the first group, the size of small holes forming the cladding was fixed at *d* = 2.2 μm, while the diameter of the two large holes, *D*, ranged from 4.2 to 5.1 μm. In the second group, the diameter of the two large holes was fixed at 4.5 μm while the sizes of small holes ranged from 1.6 μm to 2.8 μm. For all fibers in both groups, the stress induced changes in the refractive index distributions as well as the effective indexes of the LP_01-slow_ and LP_01-fast_ modes were numerically calculated at pressures ranging from 0 to 34.4 MPa.

**Figure 2.**
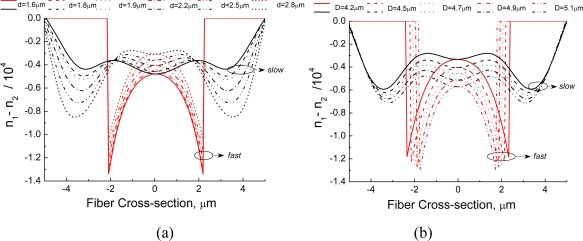
Difference between the refractive index components *n*_1_ and *n*_2_ along the slow and fast axis [see Figure 1(b)] for a hydrostatic pressure of 34.4 MPa and *λ* = 1,500 nm as function of the **(a)** large hole diameter and **(b)** small hole diameter.

Figure 2 shows, for the two groups of PCFs, distributions of the difference between *n*_1_ and *n*_2_ along both the slow and fast axis when the fibers are submitted to a hydrostatic pressure of 34.4 MPa (results are for *λ* = 1,500 nm). In Figure 2(a) one observes that by enlarging *D* and keeping *d* fixed, the absolute value of the difference *n*_1_ – *n*_2_ also increases along both axes. This indicates that stress-induced birefringence sensitivity to hydrostatic pressure, in this particular fiber design, can be enhanced by increasing the diameter of the large holes. On the other hand, variations in diameter of the small holes have a distinct effect. In Figure 2(b), we notice that when the size of the large holes is fixed and the small holes enlarged, the absolute value of the difference between indexes *n*_1_ and *n*_2_ decreases in the core region along both polarization axes.

**Figure 3.**
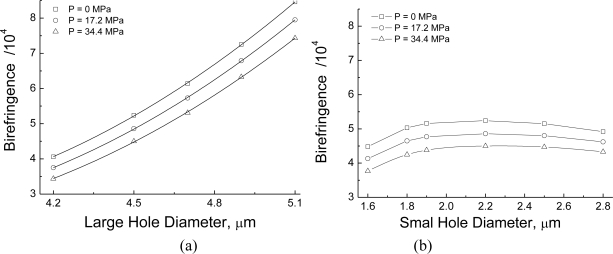
Phase modal birefringence variation with **(a)** the diameter of the large holes when *d* = 2.2 μm and **(b)** the diameter of he small holes when *D* = 4.5 μm. Lines are for eye guidance. In all cases, *λ* = 1,550 nm.

The plots in Figure 3 illustrate how the phase birefringence changes with the sizes of the large and small holes. These results were numerically obtained for different levels of the applied hydrostatic pressure and at a fixed wavelength, *λ* = 1,550 nm. As the size of the large holes increases [Figure 3(a)], birefringence rises steadily as a result of the magnification in the fiber geometric anisotropy. On the other hand, when the diameter of the large hole is fixed, birefringence initially increases as the small hole is enlarged [Figure 3(b)], but reaches a maximum value and then start to decrease. As shown in Figure 3(b), for *D* = 4.5 mm and *λ* = 1,550 nm, maximum birefringence occurs near *d* = 2.2 μm. We further observe in Figure 3 that the application of a hydrostatic pressure produces a decrease in phase birefringence for all the PCF geometries simulated here. The decrease rate, or the dependency of phase birefringence with the hydrostatic pressure, is approximately the same for all combinations of large and small hole sizes numerically investigated here.

## 3. Hydrostatic Pressure Sensor

**Figure 4.**
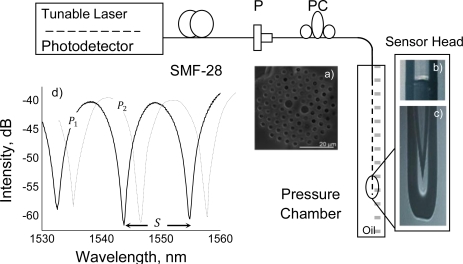
Sensor setup. SMF28: standard single mode fiber; P: polarizer; PC: polarization controller. Insets: **(a)** HiBi fiber cross section; **(b)** Optical image of the splice between the PCF and the standard fiber and **(c)** fiber end-cap; **(d)** Broadband interference spectrum indicating the space *S* between two fringes.

The sensor proposed here brings a novelty which allows it to operate in reflection while immersed in a liquid, in contrast with other PCF-based pressure sensors found in the literature that operate in transmission [1–4]. Indeed, requiring access to both ends of the fiber sensor may prove hard to implement in many practical situations, such as in petroleum wells, for instance. In our sensor, the end-face of the sensing PCF is isolated from the external medium by an end-cap made of a capillary fiber (internal hole diameter ∼56 μm), which is spliced onto the PCF fiber with the opposite end collapsed by an electric arc. Hence, Fresnel reflection at the silica/air interface at the end of the PCF is kept constant and, in addition, ingression of fluid into the PCF holes is avoided. The sensor, which is schematically depicted in Figure 4, employs the PM-1550 HiBi PCF supplied by NKT Photonics [Figure 1(a)]. According to the manufacturer, the nominal diameters of the large and small holes in this fiber are 4.5 and 2.2 μm respectively, but dimensional measurement with scanning electron microscopy provided a value of 4.1 μm for *D*. The splice loss between the SMF28 and the HiBi PCF was 2 dB, in accordance with the value obtained by Xiao *et al*. [20]. A sensor head is made by encapsulating the sensing fiber in a 1/8 inch (outer diameter) stainless steel tube filled with silicone oil. An epoxy resin is used to fix the HiBi PCF to one end of the tube while the other end is fit with a hydraulic connector for pressure intake. Light from a commercial optical sensing interrogator (Micron Optics sm125) is launched into a standard telecom fiber (SMF28) which is connected to a fiber polarizer and a polarization controller that controls the light polarization angle relatively to the HiBi PCF symmetry axis. The resulting interference over a broadband spectrum is measured in reflection by a photodetector integrated into the interrogator.

### 3.1. Modal Interferometer

The potential use of modal interferometry as a fiber sensing strategy has been early recognized when the interference between the lowest optical modes in standard fibers was demonstrated [21,22]. Due to the advantages of using PCFs in sensing applications, a number of configurations based on modal interferometers [23] have already been proposed, among other applications, for strain, temperature, and hydrostatic pressure measurements [2–4,23–27].

In this paper, an in-fiber modal interferometer is used to assemble a hydrostatic pressure sensor for which the operating principle is based on the interaction between the two orthogonally polarized modes that co-propagate through a HiBi PCF. Superposition of the two modes propagating with different phases results in a guided light spectrum showing quasi-periodic oscillations over a large wavelength range. The phase difference between the two modes, denoted as *φ* may be expressed as a function of hydrostatic pressure, *P*, the sensor length, *L*, and the wavelength:

(4) $$\varphi (\lambda ,P,L)=\frac{4\pi L}{\lambda }B(\lambda ,P)$$

Notice that the modal birefringence depends on the wavelength and also on the applied pressure. Furthermore, since the interferometer was assembled in reflection, the optical path length is. 2*L*.

The broadband interference spectrum is illustrated in Figure 4(d). The dips correspond to those wavelengths where the phase difference between the two polarizations are integer multiples of. 2*π*. The phase difference changes with wavelength, applied pressure, and the optical path length as follows

(5) $$\begin{array}{c} \Delta \varphi =\frac{\partial \varphi }{\partial \lambda }\Delta \lambda +\frac{\partial \varphi }{\partial P}\Delta P+\frac{\partial \varphi }{\partial L}\Delta L\\ =-\frac{4\pi L}{\lambda^{2}}\left(B-\lambda \frac{\partial B}{\partial \lambda }\right)\Delta \lambda +\frac{4\pi L}{\lambda }\frac{\partial B}{\partial P}\Delta P+\frac{4\pi LB}{\lambda }\frac{\Delta L}{L}\\ =-\frac{4\pi LG}{\lambda^{2}}\Delta \lambda +\frac{4\pi L}{\lambda }\frac{\partial B}{\partial P}\Delta P+\frac{4\pi LB}{\lambda }\frac{\Delta L}{L} \end{array}$$

Temperature effects were neglected here due to the low temperature sensitivity of this particular fiber.

The group birefringence may be related to the gap between two consecutive dips in the interference spectrum, denoted by *S* in Figure 4(d). In order to do so, we first observe that if the optical path length is unchanged (Δ*L* = 0) and the pressure level kept constant, Equation (4) reduces to:

(6) $$\Delta \varphi =-\frac{4\pi \mathrm{LG}}{\lambda^{2}}\Delta \lambda$$

By assuming that phase difference between the two polarized modes changes linearly with the wavelength, an approximation that has been often employed in the literature [3,4,6,26,27], and observing that Δ*λ* = *S* corresponds to a change in wavelength Δ*φ* = 2*π*, we may write:

(7) $$G\approx -\frac{{\bar{\lambda }}^{2}}{2\mathrm{LS}}$$

where *λ̄* is an average wavelength at the spectral range where *G* is being evaluated. Equation (7) allows us to estimate modal group birefringence from measurements of the gap between two consecutive dips in the interference spectrum.

As illustrated in Figure 4(d), the broadband interference spectrum moves along the wavelength axis when the applied hydrostatic pressure changes. Pressure is then measured by following wavelength shifts of one of the dips in the spectrum. Thus, as the pressure increases, the wavelength corresponding to one of the dips changes by an amount Δ*λ*. The phase difference between the two modes is still 2*π*, hence Δ*φ* = 0. Now, returning to Equation (5) and again considering Δ*L* = 0, we obtain:

(8) $$K_{P}=\frac{\partial B}{\partial P}=\frac{G}{\lambda }\frac{\Delta \lambda }{\Delta P}$$

Here, we have neglected the longitudinal strain in the fiber (*ε* = Δ*L*/*L*) produced by the hydrostatic pressure. Equations (7) and (8) will be used in the next section to compare experimental and simulated results.

#### 3.2.1 Results and Discussion

**Figure 6.**
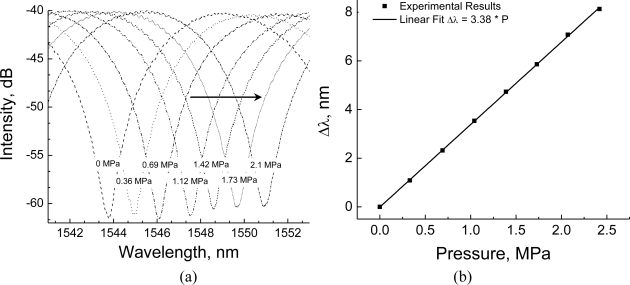
**(a)** Variations in the spectrum of the modal interferometer due to the applied hydrostatic pressure ranging from 0 to 2.42 MPa; **(b)** Typical calibration curve at constant temperature (º25 °C). Results are for *λ* = 1,550 nm and L = 143 mm.

In order to characterize its response to hydrostatic pressure, the sensor was placed in a pressure chamber immersed in a temperature calibration bath filled with silicone oil. Temperature stability of the bath was better than ±0.05 °C, therefore all variations in response where due solely to applied pressure. Figure 6(a) presents broadband interference spectra measured at different pressure levels (0 to 2.42 MPa) and at a fixed temperature of 25 °C. We clearly notice the shift in spectrum as pressure increases.

A typical calibration curve for the sensor is reproduced in Figure 6(b), which presents wavelength changes of one of the dips in the interference spectrum. There is an apparently linear dependence with hydrostatic pressure, with sensitivity of 3.38 nm/MPa. This result was obtained for a sensor 143 mm long. Sensors with six different lengths ranging from 60 to 180 mm were also tested, and the resulting sensibility, as expected from Equation (8), did not vary beyond the experimental uncertainties. The average sensibility for calibrations with different sensor lengths was found to be 3.4 ± 0.04 nm/MPa, a value very close to the one previously reported in the literature for this fiber, 3.5 nm/MPa [3].

**Figure 7.**
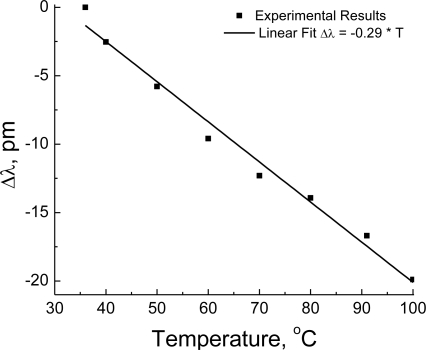
Temperature response (λ = 1,550 nm, L = 143 mm).

Temperature response was investigated by placing the sensor, unpressurized, in an oven with controlled temperature (±0.05 °C). Figure 7 presents the wavelength shifts of one of the dips in the spectrum obtained for measurements at temperatures ranging from 30 to 100 °C. Measured sensitivity to temperature was 0.29 pm/°C, a value which is in agreement with results previously reported in the literature for this particular fiber [2,3].

The average value for the measured spectral distances between two consecutive dips in the interference spectrum of the sensor built with a length of 143 mm was *S* = 11 nm. Through a simple calculation performed via Equation (7), the group birefringence at *λ* = 1,550 nm was found to be. *G* = −7.6 × 10^−4^. This value was further validated by measuring the differential group delay between the two modes, LP_01slow_ and LP_01fast_. At 1,550 nm, the measured delay was 0.39 ps, which corresponds to a group birefringence. *G* = −7.8 × 10^−4^. Both values can be considered equal within the experimental error. Now, by applying Equation (8) while considering Δ*λ*/Δ*P* = 3.4 nm/MPa, *λ* = 1,550 nm, and, *G* = −7.7 × 10^−4^, the latter being an average value from both independent measurements of the group birefringence, we obtain *K_P_* = −1.7 × 10 ^−6^ MPa ^−1^.

**Figure 8.**
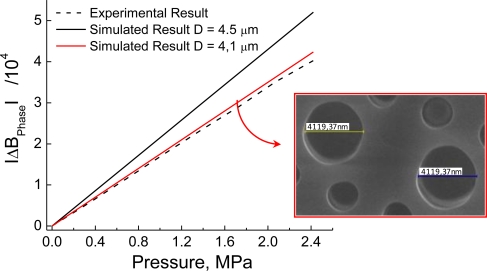
Change of modal birefringence with pressure at λ = 1,550 nm (numerical and experimental). The inset shows a microphotography of the commercial HiBi PCF used to assemble the pressure sensor.

The sensibility of modal birefringence to hydrostatic pressure may also be calculated through the numerical model discussed in Section 2. Simulations were performed at a fixed wavelength and different levels of the applied pressure. Numerical results are plotted in Figure 8, which also presents the experimental estimate of the phase birefringence variation with pressure, where *K_P_* was taken as −1.7 × 10^−6^ MPa^−1^. Numerical results were evaluated considering both the nominal dimensions of the fiber, as specified by the manufacturer, and those measured using electron microscopy. The most significant difference was found in the diameter of the large holes, specified as 4.5 μm in the supplier’s data sheet and measured at 4.1 µm (see the inset in Figure 8). The agreement between experimental and numerical results is excellent, validating the model discussed in Section 2.

One last issue concerns single-modeness in PCFs, which is a consequence of the large leakage losses exhibited by their higher order modes. It should be considered, however, that the length of fiber plays a fundamental role in that regard. Indeed, a detailed study on the modal content in a fiber that can be considered as being endlessly single mode for long propagation distances has shown that the presence of higher order modes in short fiber lengths can also give rise to modal interference [28]. In the scheme employed to interrogate our pressure sensor, modal interference of higher order modes would appear as a noisy signal superimposed to the pattern generated by the beating of the two fundamental ones. However, for the sensing lengths of the prototypes tested in the present investigation, which ranged from 60 to 180 mm, the signals were fairly noise-free. This indicates that the interaction between the two orthogonally polarized fundamental modes dominates the resulting interference spectrum. Furthermore, since the intermodal interference between the higher other modes would appear as a short period oscillation in the spectrum, proper signal processing could eliminate such an effect and, in principle, allow the implementation of much shorter sensing lengths.

## 4. Conclusions

Numerical modeling was used to study the behavior of the optical modes confined within HiBi PCFs under hydrostatic pressure. By using a reference design provided by a commercially available PCF as a starting point, and producing slight geometrical modifications by independently changing the diameter of their small and large air holes, we have evaluated effects of geometry on the sensibility of modal birefringence to hydrostatic pressure. It was found that the difference between the refractive index components *n*_1_ – *n*_2_ along the slow and fast axis of the fiber increase as we enlarge the diameter of the larger hole. On the other hand, the difference *n*_1_ – *n*_2_ decreases within the fiber core and increases in the cladding region as the small holes are enlarged. We have also shown that phase modal birefringence is enhanced by increasing the size of large holes. The numerical results were compared with experiments showing excellent agreement.

A pressure sensor using a HiBi-PCF as the sensing element and an in-fiber interferometric scheme for interrogation was proposed and demonstrated. The sensitivity to hydrostatic pressure was estimated to be 3.4 nm/MPa, while temperature sensitivity was much lower, only 0.29 pm/°C. This means that a variation of 100 °C could be interpreted as an apparent pressure change of 8.5 × 10^−3^ MPa, an error that may be acceptable in some applications, depending of course on the operating pressure and temperature ranges as well as on the required accuracy in pressure measurement. For a given application, if this error is indeed acceptable, the simultaneous use of a temperature sensor for compensation may then be unnecessary. This is certainly an advantage of PCFs over other competing fiber optic sensor technologies.

Although the sensor was tested only up to 2.5 MPa and 100 °C, its operational range will be limited mainly by the sealing capability of the encapsulation. The fiber itself is capable of withstanding much higher pressures and temperatures. The interrogation scheme, however, may present an additional limitation to the pressure range of the sensor. The broadband interference spectrum is periodic, and a strategy to implement continuous pressure monitoring would be to limit the excursion of the spectrum to one period, which corresponds to the distance between two consecutive dips in the spectrum. Equation (7) shows that the sensor length and the distance between two consecutive dips are inversely related, *i.e.*, the sensor length must be shortened in order to increase. *S.* A simple calculation employing the results obtained in the paper indicates that, in principle, by using a sensor 5 mm long, the distance between two consecutive dips in the spectrum near 1,550 nm would reach 312 nm. Considering the estimated sensibility of 3.4 nm/MPa, an excursion of one period in the spectrum would correspond to a pressure of 92 MPa, a satisfactory operating limit for a number of industrial applications. Considering that the current technology of tunable laser interrogators provides wavelength resolutions that are better than 1 pm, the resolution of the proposed sensor can be estimated at 3 × 10^−4^ MPa. For a sensor with an operating limit of 92 MPa, this corresponds to 0.0003% of full scale resolution. However, the issue of noise induced by higher order modal interference should be taken into account when designing such a short sensor. If not properly addressed, by, for instance, filtering the high-frequency oscillations in the spectrum, the beating of higher order modes could prevent the implementation of sensors whose sizes are shorter than a few centimeters.
